# Adaptation of the Spanish model of organ donation: experience from a public hospital in Argentina

**DOI:** 10.62675/2965-2774.20260264

**Published:** 2026-01-28

**Authors:** Nicolás Sebastián Rocchetti, Julián Pablo Juárez, Ender Azuaje, Pablo Centeno

**Affiliations:** 1 Hospital Eva Perón Intensive Care Unit Granadero Baigorria Santa Fe Argentina Intensive Care Unit, Hospital Eva Perón - Granadero Baigorria, Santa Fe, Argentina.; 2 Hospital de Alta Complejidad del Bicentenario Esteban Echeverría Intensive Care Unit Monte Grande Buenos Aires Argentina Intensive Care Unit, Hospital de Alta Complejidad del Bicentenario Esteban Echeverría - Monte Grande, Buenos Aires, Argentina.

## INTRODUCTION

The Spanish model of organ donation and transplantation has indisputably become the gold standard to follow, due to its results and success in sustaining high donation and transplantation rates. The model is characterized by a three-level management structure within a public health system and a network of hospital coordinators with a special profile, added to a hospital reimbursement strategy, press attention programs, and a significant investment in training, in the context of a committed and supportive society.^(1)^

One of the keys to the Spanish model is the figure of the hospital transplant coordinator, who, in the vast majority of cases, is represented by intensivists (the backbone of the donation process), who, in addition to working as such, also serve as part-time hospital coordinators.^(2)^ Argentina and other countries adopted this model years ago, with favorable results,^(3)^ positioning Argentina as a leader in Latin America.^(4)^ However, in specific contexts, adaptations may be required.

We aimed to evaluate the impact of implementing an adaptation of the classic Spanish donation model (without a hospital transplant coordinator) in a hospital of a middle-income country (Argentina).

## METHODS

This was a retrospective, single-center, observational, analytical study at a highly complex public hospital (*Hospital del Bicentenario de Esteban Echeverría* [HBEE]) with 140 beds and a 27-bed intensive care unit (ICU), admitting 600 patients/year and with a mortality of 24%. Its reference population is approximately 3 million people shared with another high-complexity hospital, and since its inauguration in 2020, it has generated donors (with this adapted model). Data were obtained from the *Central de Reportes y Estadísticas* (CRESI) of the *Sistema Nacional de Información de Procuración y Trasplante de la República Argentina* (SINTRA), a public, anonymized database that does not require informed consent or additional ethical clearance.^(5)^

*Hospital del Bicentenario de Esteban Echeverría* does not have a transplant coordinator, but almost the entire donation process is handled by the ICU intensivists, from the detection of the potential donor, through the diagnosis of brain death, selection, family communication, and judicial authorization if necessary, to the treatment of the donor, leaving the logistics of organ and tissue distribution to the relevant organization.

The primary outcome was the number of organ donors in 2024, compared to the two hospitals in Argentina that generated the most donors after the HBEE and the hospital in Spain that generated the most donors in 2023. Chi-squared test was used to compare categorical variables.

## RESULTS

Since its opening in 2020, HBEE has progressively increased donation activity, reaching 84 organ donors in 2024 (70 in brain death and 14 after circulatory death), representing 10.04% of all cadaveric organ donors in Argentina that year (n=837). This number was significantly higher than those of the second and third hospitals in Argentina (54 donors and 25 donors, respectively; p < 0.001 for both).^(5)^ The figure was comparable to the leading hospital in Spain, Virgen de la Arrixaca Clinical Hospital, with 92 donors in 2023 (p = 0.39)^(4)^ (Table 1).

**Table 1:** Number of organ donors at *Hospital del Bicentenario de Esteban Echeverría* in 2024 compared with the two following hospitals with the highest number of donors in Argentina (2024), and the leading Spanish hospital in 2023 (Virgen de la Arrixaca Clinical Hospital, El Palmar)

Hospital	Country	Year	Donors (n)	p value (versus HBEE)
HBEE	Argentina	2024	84	Reference
2nd highest in Argentina	Argentina	2024	54	< 0.001
3rd highest in Argentina	Argentina	2024	25	< 0.001
Virgen de la Arrixaca	Spain	2023	92	0.39

HBEE - *Hospital del Bicentenario de Esteban Echeverría.*

Data from the *Central de Reportes y Estadísticas* (CRESI) of the *Sistema Nacional de Información de Procuración y Trasplante de la República Argentina* (SINTRA), and the Global Observatory on Donation and Transplantation (GODT).

## DISCUSSION

Our results suggest that it is feasible to sustain high donation rates in a middle-income setting without a formal hospital transplant coordinator, with intensivists leading the process. This represents an adaptation of the Spanish model rather than a new paradigm, reinforcing the central role of intensivists already recognized in the reference model.^(2)^

Limitations include the retrospective and single-center design. Therefore, these findings should be considered hypothesis-generating and require confirmation in multicenter studies. Nevertheless, the experience at HBEE illustrates that, in specific contexts, donation can be successfully organized around ICU leadership, supporting intensivists as the backbone of the process.

## Data Availability

The contents underlying the research text are included in the manuscript.
